# INTERVENTIONS FOR FAMILY CAREGIVERS RECEIVING PALLIATIVE/HOSPICE CARE AT HOME: A SCOPING REVIEW

**DOI:** 10.1093/geroni/igac059.3081

**Published:** 2022-12-20

**Authors:** Taeyoung Park, Sulaiman Alshakhs, Elisabeth Sweet, Meghan McDarby, Sara Czaja, Cary Reid, Ronald Adelman, Veerawat Phongtankuel

**Affiliations:** Weill Cornell Medicine, New York, New York, United States; Weill Cornell Medicine, New York, New York, United States; Weill Cornell Medicine, New York, New York, United States; Memorial Sloan Kettering Cancer Center, New York, New York, United States; Weill Cornell Medicine, Ithaca, New York, United States; Weill Cornell Medicine, Ithaca, New York, United States; Weill Cornell Medicine, Ithaca, New York, United States; Weill Medical College of Cornell University, New York, New York, United States

## Abstract

With the growth of palliative and hospice care, patients with a terminal illness and their family caregivers have opportunities to pursue care focusing on alleviation of symptoms and optimizing quality of life in the home setting. However, caregivers are often overburdened with caregiving responsibilities leading to poor physical and mental health. Given the importance of family caregivers for patients who elect to receive hospice/palliative care at home along with the negative outcomes associated with caregiving, conducting a review of caregiver interventions is critical to understand what has been studied and identify gaps in the field. A scoping review was conducted to describe interventions focused on family caregivers providing care to terminally ill patients who receive palliative/hospice care in the home setting. Variables collected included intervention delivery details, study population, participant demographics, and measured outcomes. Our search yielded 14,527 articles, of which 70 articles on 54 unique interventions were included in the final analysis. Over three-quarters (77.8%) of the interventions were conducted in North America and Europe. Interventions commonly aimed to reduce the physical burden of caregiving (53.7%), educate the caregiver (53.7%), and improve psychological well-being (48.1%). The majority (58.6%) of the articles evaluated interventions for feasibility outcomes. The lack of studies in non-Western countries points to a cultural disparity in this field. Secondly, despite the variety of interventions, religious and spiritual needs in palliative care need to be further addressed. As palliative and hospice care is an emerging area of research, most studies are still implementing interventions and assessing feasibility.

